# Exploring the opportunities and challenges of female health leaders in three regional states of Ethiopia: a phenomenological study

**DOI:** 10.1186/s12889-022-13871-w

**Published:** 2022-08-02

**Authors:** Sualiha Abdulkader Muktar, Binyam Fekadu Desta, Heran Demissie Damte, Wubishet Kebede Heyi, Elias Mamo Gurmamo, Melkamu Getu Abebe, Mestawot Getachew Mesele, Mesele Damte Argaw

**Affiliations:** USAID Transform: Primary Health Care, JSI Research & Training Institute, Inc. in Ethiopia, P.O. Box 1392 code, 1110 Addis Ababa, Ethiopia

**Keywords:** Leadership, Female leaders in health, Career path, Opportunities and challenges, Ethiopia

## Abstract

**Background:**

Gender equity involves fairness in all aspects of life for women and men and is usually determined by social, political, economic, and cultural contexts. The proportion of female leaders in healthcare within the health sector is low. The aim of this study was to explore and describe the experiences, opportunities, and challenges faced by women in their path towards becoming leaders within the health sector.

**Methods:**

This study was conducted using the phenomenological method of qualitative inquiry. The approach was chosen for its merits to narratively explore and describe the lived stories and shared experiences of women leaders in the healthcare system. A purposive sampling technique was used to identify six women leaders. Semi-structured interviews were conducted through telephone by the investigators. The qualitative data analysis was conducted parallel with data collection, using steps of thematic analysis.

**Results:**

This study identified individual, societal, and organizational level opportunities and challenges that had an influence on the career paths of female health leaders in Ethiopia. The leadership positions were an opportunity in the career development of women who had long-term goals, were known for their empathy, and exercised wise use of resources. In addition, women who had the support of close family members and their peers are more likely to compete and rise to leadership positions. Furthermore, women who received organizational support in the form of affirmative action, training, development, and recognition also tended to rise to leadership positions. However, women who assumed leadership positions but whose day-to-day decision-making was influenced by their supervisors, those who had experienced sexual harassment, and those under the influence of societal norms were less likely to attain leadership positions.

**Conclusion:**

The opinions and experiences of female health leaders revealed that individual behaiour whileassumming a leadership positon, empathy, and wise resource management positivey influence their career development. In addition, female health workers who had support form close family members and peers strived for growth to leadership positions. Furthermore, the presence of organizational support, in the form of affirmative actions, and succession planning were another opportunity for females in their career paths. Conversely, some social norms were found to deter female health workers from advanicing to leadership positions. Therefore, enhancing the leadership capacity of women and improving social and organizational support is recommended. In addition, addressing the low level of self-image among women and patriarchal societal norms at the community level is recommended.

**Supplementary Information:**

The online version contains supplementary material available at 10.1186/s12889-022-13871-w.

## Background

Globally, slightly lower than three fourths of the health and social sector workforce are assumed by women [1]. Women are the main providers of health to around five billion people and they contribute $3 trillion annually to global health. Approximately half of this contribution is in the form of unpaid care work [2]. The progress towards ensuring gender equality in health leadership and management positions are affected by systemic challenges and gender biases. These challenges have been linked to health system inefficiencies [3]. Therefore, inequities persist in the health leadership⁠—predominantly to the disadvantage of women [4–7].

Leadership and governance (stewardship) is one of the critical elements of the conceptual framework proposed to strengthen the health system of any country by of the World Health Organization (WHO) [8, 9]. In the last two decades, many organizations have strived to build desired comptencies to lead, manage, and govern effectively and efficienty, applying the five components of health system building blocks; namely, service delivery; health workforce; health information system; medical products, vaccines and technologies, and financing [8]. In addition, these interventions are believed to create a resilient and responsive health system.

Gender equity involves fairness in all aspects of life for women and men and is usually determined by social, political, economic, and cultural contexts [10]. However, studies conducted in Australia and Ethiopia confer that the societal norms in favor of men are deep-rooted in the community and influence women’s self-image and aspirations from childhood to adulthood [11, 12]. In addition, this unfavorable environment has become a challenge to women’s career development and is demonstrated by fewer representations of women in senior professional and leadership positions [13]. It was found for example that the career progression of female health workers in Nigeria and Egypt were disrupted due to their additional family duties [14–16]. Meanwhile, the career development of UK and US female health wokers was found to have been disrupted due to unfavourable work environments [14]. Furthermore, the societal norms discouraged female health workers from progressing in their leadership careers [17, 18].

A great number of influences have shaped the leadership opportunities available to women in the healthcare sector overtime. Although many countries acknowledge that the existence of gender norms in leadership positions are in favor men rather than women, there is little evidence of efforts and investments being applied to change the situation [19–22]. Therefore, this study aims to explore and describe the experiences, opportunities, and challenges faced by women in their path towards becoming leaders within the health sector, in three regional states of Ethiopia.

## Materials and methods

### Study design

For this study, the phenomenological method of qualitative inquiry was used [23–25]. This approach was chosen for its merits to narratively explore and describe the lived stories and shared experiences of women leaders in the healthcare system [23]. The data were collected from March–July 2021.

### Study setting

Ethiopia is located 3′ and 14.8″ latitude 33′ and 48′ longitude in the horn of Africa. The decentralized federal state consists of eleven regional states and two city administrations. The health system is made up of three tiers that contain 17,550 heath posts, 3735 health centers, and 353 hospitals. Within these health facilities, there are 275,899 health workers and 54% of the workplace positions are occupied by women. Among these only 1.0% of women, hold healthcare leadership positions [26].

The USAID Transform: Primary Health Care project provides leadership, management, and governance (LMG) training and interventions in six regional states of Ethiopia, namely: Amhara, Oromia, Sidama, Southern Nations, Nationalities, and Peoples’ (SNNP), South West Ethiopia, and Tigray. During the last four years of the project’s implementation period (2017-2020), a total of 2682 health workers, recruited from 644 primary healthcare entities were capacitated on LMG competencies. Of the trained health workers, 15% were women leaders in health, managers, or staff from primary healthcare units. In addition, three ‘women only’ LMG cohorts were formed, and 78 female health workers were trained in Amhara, Oromia, and SNNP regions. Within the cohorts of LMG trained health workers, 18 were promoted to senior leadership positions within the primary healthcare system [12].

### Population and sampling

The target population for this study was health leaders that are women, working at primary healthcare entities. A purposive sampling technique was employed to identify the targeted participants, with a pre-defined criterion that selected women healthcare leaders that are working within the various primary health system levels in three regional states of Ethiopia.

### Data collection

Data were collected using the iterative process of in-depth individual interviews. The English version in-depth interview guides developed by Javadi et al., (2016) [10] were directlry adapated for this study and then translated into Amharic and Afan Oromo languages. To maintain the consistency of the tools it was translated back to English by the investigators. The interviews were conducted using local official languages of the study areas, i.e. Amharic and Afan Oromo. Semi-structured telephone interviews were conducted by the female investigators which also captured the participants’ socio-demographic information [10]. The two main question were: ‘(1) what have you experienced as a woman on your path towards health leadership?’, and ‘(2) what contexts or situations have typically influenced or affected your experiences as a woman in a leadership position?’. These questions were helpful to explore the phenomenon and experiences of women in the contexts of assuming leadership positions. Probing questions were applied and were used to uncover the individual, household, organizational, economic, and political opportunities and challenges faced by women leaders in healthcare (Additional file 1). On average, each in-depth interview lasted about 60 minutes. Digital recorders and handwritten notes were used to capture the information gathered from the in-depth interviews. After six indepth interviews, the data collection process was concluded in line with the recommendation of Body (2001) [27].

### Data analysis

The data analysis was conducted using the convergent parallel approach to data collection [28]. The audio recorded data were transcribed verbatim on the same day by the investigators and were reappraised with the interviewees for accuracy. The transcripts were then translated from local languages into English by the interviewers. Four independent investigators read and re-read each transcript and became familiar with the data. A woman’s unique experience in the journey towards achieving a leadership position in health was identified and the investigators looked for common themes across stories. Quotes were extracted and collected under identified codes. The investigators held consensus discussions on the codes and examined the meaning of equal values. Once the codes were defined, the data analysis was continued until all themes and categories were identified. Since this study follows a phenomenological inquiry, the research participants were engaged at all stages of the research activities. Hence, participants reviewed transcripts, provided their comments on the preliminary findings, codes, categories, and themes, and finally approved highlighted quotations and descriptions [29].

### Inclusion criteria

- Familiar with the different components of the primary healthcare system.

### Measures to ensure trustworthiness

This study was led by LMG experts with experiences in qualitative inquiry. The data collection guides were piloted. The lived stories, shared experiences, and perceptions of the female leaders in healthcare were narratively collected. The trustworthiness of this study was ensured through maintaining four criteria, namely: credibility, transferability, dependability, and confirmability [30]. The credibility of this study was maintained through the prolonged engagement of investigators and reviewing of transcripts by respondents during the data collection process, analysis, and interpretation. The transferability of this study was ensured through the dense description of the research methods, particular phenomena, and the contexts of this research activity [29]. The dependability of this study was maintained through an in-depth description of the steps employed for the adopted research method. Finally, the confirmability of this research’s findings was ensured through the active participation of respondents in the processes of data collection, analysis, and interpretation [31].

### Ethical considerations

This study protocol was carried out in accordance with the Declaration of Helsinki and ethical clearance was obtained from John Snow Inc. institution review board (IRB) with certificate ref. no. IRB #21-06E. Before starting data collection, permission and support letters were collected from regional state health bureaus. After providing a brief orientation on the purpose of the study, an informed written consent was obtained from each participant. All participants were adults i.e., greater or equal to 18 years, and had the right to discontinue or refuse to participate in the study at any time. MDA has received the behavioral and social research ethics online training at the Collaborative Institutional Training Initiative (CITI) program and oriented all investigators on the need to maintain ethical principles like privacy, anonymity, and confidentiality during data collection, analysis, and report writing activities.

## Results

### Participant characteristics

This qualitative phenomenological study was conducted through in-depth telephone interviews with six female leaders in health in three regional states of Ethiopia. The educational background of all participants were linked to bachelors of sciences in health studies. The average overall services tenured by the participates was nine years. However, the participants had been in leadership positions for about two years (Table 1).

**Table 1:** Participant descriptions

Study participant ID	Leadership position	Age	Overall work experience in years	Years in leadership position	Educational background	Leadership training
ID1	Clinical services auditor	29	Four	One	BSc in public health	Yes, off-site six-day classroom basic LMG training followed by coaching for nine months.
ID2	Head of health center	39	Nineteen	One	BSc in midwifery	Yes, off-site six-day classroom basic LMG training followed by coaching for nine months.
ID3	Pharmacy head	28	Six	Two	BPharm	Yes, off-site six-day classroom basic LMG training followed by coaching for nine months.
ID4	Primary health care unit director	31	Six	One	BSc in public health	Yes, six-day classroom basic LMG training and nine months’ practical on-the-job experience.
ID5	Deputy head of district health office	31	Eleven	Three	BSc in public health	Yes, off-site six-day classroom basic LMG training followed by coaching for nine months.
1D6	Head of district health office	27	Eight	Four	BSc nurse	Yes, off-site six-day classroom basic LMG training followed by post training coaching and support for nine months. She also received leadership training for a month while on the leadership position.

The table presents the socio-demographic characteristics of the research partcipants

The qualitative data analysis revealed four themes and thirteen categories (Table 2).

**Table 2:** Themes and categories

Themes	Categories
1. Individual behavior	1.1. Being visionary 1.2. Empathy and honesty 1.3. Wise use of resources 1.4. Leadership experience
2. Social support	2.1. Family support 2.2. Peer support
3. Organizational support	3.1. Affirmative actions 3.2. Training and mentoring 3.3. Succession planning 3.4. Recognition 3.5. Development partners’ support
4. Gender stereotype (cultural norms/ societal factors)	4.1. Status quos and norms 4.2. Self-image

The table depicts the identified themes and categories from qualitative data analysis

### Theme 1: individual behavior

This theme emerged from four categories of the data analysis results. The participants of this study described individual behaviors such as being a visionary person, an empathetic listener, honest, and applying wise use of limited resources as making a woman an ideal candidate for a leadership position within primary health facilities. In addition, the participants described the importance of having experience in leadership for achieving better results in work-family life.

### Category 1.1: being visionary

The research participants explained that a leader with a clear vision has the potential to achieve better results and they tend to self-initiate their rise to leadership positions in their organizations. The following extract which is verbatim, illustrates the commitment of one visionary woman on her path towards a leadership position in health.

I want to be successful in my educational career and in business too. It is my dream to be a successful healthcare leader. I am sure one day I will serve as a role model for young girls in my vicinity. Participant #1.

### Category 1.2: empathy and honesty

Participants of this study stated that female leaders in health spent a lot more time on listening to their staff than their cpunterpart male colleagues. This behavior forsters an opportunity for women to progress in their path towards leadership and management positions. Participant #3 described her efforts towards building a culture of honesty in the Ethiopian health system:

I am trustworthy and honest with my staff and organization. I ensure that there is no corruption in the health center. Therefore, I am among a few effective health leaders. That is why I have managed to hold on to a leadership position, even in difficult times. Participant #3.

### Category 1.3: wise use of resources

Participants perceived that women are more likely to engage in the wise use of health facility resources. This behavior made women the preferred health system leaders. Participants #5 and #6 described how this behavior has assisted them to rise to leadership positions.

Almost all women have experience in managing and leading their households. A woman can easily identify the needs of her child and can properly address it in the best way …. these management practices and experiences capacitate women leaders to use available resources efficiently in health facilities too. Participant #5.

### Category 1.4: leadership experience

In this study, participants stated that women who had experiences of leadership were more likely to volunteer to hold leadership positions in their organizations. A participant illustrates how her experience influences her current role in a leadership position.

I was a health extension worker supervisor, students’ representative at university, and have undergone leadership training; all of which have enriched my experiences. I am a strong woman by nature. Growing up in a rural area with a lot of problems helped me become resilient and cope with challenges easily. By using my experience I manage my day-to-day activities very well and my health center’s performance is very good. Participant #4.

Despite this, there are challenges which negatively impact women’s ascension to leadership positions at primary healthcare entities in Ethiopia. In this study, participants described their lived stories of reluctance in delegation of leadership positions combined with micromanagement by supervisors as barriers. In addition, experiences of workplace sexual harassment were reported as a challenge for the women in their rise to leadership positions. A woman leader describes these challenges as follows:

Previously, officials used to give authority to female leaders but would be deeply involved in the execusion of their responsibilities. Because I was having these discouraging experiences, I would often avoid putting myself up for leadership positions. In addition, I know cases of sexual harassment that female leaders encountered. It not only affects their work, but can also impact their private family life. I have missed a few opportunities because I was afraid of going through similar ordeals. Participant #2.

### Theme 2:- social support

Participants frequently described the positive effects of support from spouses, grandparents, and colleagues in their path towards leadership positions. The theme social support was identified from close family and peer support categories.

### Category 2.1: close family support

Almost all participants of this study mentioned that women who had received support from their spouse and close family members are more likely to advance to leadership positions. Participant #6 describes the support of her close family members a follows:

My husband encourages and supports me in my leadership career development. I had discussed the leadership opportunity at my organization with my spouse and got his input before I decided to compete for the position. Participant #6.

Similarly, some participants of this study frequently stated that women who had the burden of household chores and who do not have support of close family members would avoid assuming leadership positions. A female leader recalls her experience of avoiding leadership positions due to lack of family support by saying:

After I had two children, my mother and my mother-in-law advised me to leave my job and concentrate on household chores. I was responsible to taking care of my children, cooking meals and feeding them, washing clothes, and managing all other aspects of family life. … .for a woman like me, leaving children with their father or other family members for a long periods of time was not possible and therefore, I declined opportunities that came my way. Participant #5.

### Category 2.2.: peer support

In this study, participants linked their preparedness for leadership roles to the support they received from their supervisors which were expressed as trainings, mentorship, coaching, and feedback. Participants #3 and #5 expressed their positive experiences with peer support as follows:

…when I was a family health department coordinator, [a colleague] supported me to build my confidence in handling more than my current responsibilities. Before I assumed my current post, I discussed it with my best friend and it is because of her encouragement that I took on the leadership position. Participant #5.

On the otherhand, participants attributed lack of peer support as the main reason for women avoiding leadership positions. Participant #1 explains that lack of peer support negatively influenced her leadership career development saying:

My colleagues laughed at my decision to assume a leadership position while having children who need my full-time care and they discouraged me to continue my career development. Participant #1.

### Theme 3:- organizational support

Leadership development is a process of capacitating health workforces while adhering to principles and guidelines of an organization. The theme organizational support emerged from four categories, namely: principles and guidelines, training and mentoring, succession planning, and development partners’ support.

### Category 3.1: affirmative action

All participants identified and explained the importance of adherence to principles and guidelines in increasing female leaders in health organizations. Clear principles and guidelines from health leaders solve complaints from staff and simplify activities. Participant#1 illustrates the importance of adherence to guidelines as follows:

The human resources manager follows pre-defined guidelines in the processes of selecting the best candidates for advertised posts. Though some candidates raised issues of fairness on the selection processes, the core process owner was able to check for consistency and reliability on the points given to each participant. Participant #1.

### Category 3.2: training and mentoring

In this study all participants attested to feeling more capable following leadership training and mentoring sessions. The following participant stated the benefits of leadership training in capacitating women health leaders by saying:

The leadership training in which I took part in helped me to cope with challenges I faced. Furthermore, while I was implementing my project, the coaching sessions helped me adapt some revisions that had been made on leadership, management, and governance practices, experience sharing, and developing doable actions. Participant #1.

### Category 3.3: succession planning

In this study, participants explained that women who were identified as future leaders by their immediate supervisors and got prepared to face new challenges before assuming leadership positions were successful in their career development. Participant #3 describe the benefits of engaging and preparing women for leadership positions in advance, as follows:

In my current organization, my immediate supervisor encourages me to build my confidence and courage, and practice my leadership and management skills. He always delegates me when he has other commitments outside. This has a huge impact on my self improvement and in helping others achieve better results. I am also really motivated to work towards leadership when my supervisor delegates me, and the support from my colleagues (especially women) helps me recognize my shortcomings and enables me to work on them. Participant #3.

### Category 3.4: recognition

To encourage more women to assume leadership positions, the health system should recognize and motivate the role models in the health system. In this study, participant #2 explains the importance of exercising recognition for best performing women in increasing leaders.

…to attract women to leadership positions, there should be incentives and recognition like offering continuous education. Participant #2.

### Category 3.5: development partners’ support

All participants stated that their leadership capacity, competencies, and capabilities were enhanced with the support of development partners. This verbatim of participant #6 shows the role of development partners on women’s leadership development:

I thank [development partner name] for their support. I took leadership training which prepared me for my day-to-day activities in leadership. Participant #6.

### Theme 4.: gender stereotypes

Gender stereotypes are the generalized views of the community about the roles played by men and women. These held beliefs can affect how women assume leadership positions. Gender stereotyping was identified from two categories i.e. status quos (norms) and self-image.

### Category 4.1: status quos and norms

All six participants stated societal norms hinder women from assuming leadership positions. The following are verbatims from participants #1 and #6, which summarize the opinions of the rest of the respondents.

In my district, the social norms favor men and there is limited support for women to progress in their career development. Women are expected to manage all household matters and are perceived as weak in leading organizations. Participant #1.

…community members including trained professionals think of women as too weak to lead complex organizations. The workload of a leadership position, a busy home life, and the expectation of staff can sometimes create frustration. Participant #6.

### Category 4.2: self image

In this study, four out of six participants explained that lack of self-image and confidence are among reasons for women being hindered in their leadership career development. Participant#5 states:

…I consider myself as weak and someone who cannot handle a lot of responsibilities. This is why I declined leadership positions three or four times due to the fear of failure. Participants #5.

## Discussion

Many studies have documented the significant contributions of female leaders in high performing organizations. This phenomenological qualitative inquiry explored and described the lived stories and shared experiences of six female health leaders in Ethiopia. The data analysis identified four themes; namely: individual behaviour, social support, organizational support, and gender stereotypes. The first theme which emerged from categories consists of being visionary in leadership career development, empathy, resource management, and leadership experiences. Close family and peer support were subcategoties for the second theme, social support. The third theme was identified through five sub categories, namely: affirmative actions, training and mentoring, sucession planning, regognition, and development partners’ involvement. The fourth theme, gender steropype, emerged from social norms and self image sub categories. The findings of this study will hopefully influence policy makers, program managers, development partners, and health workers in the future in improving efforts towards increasing female leaders and managers in health.

According to the participants, the individual behavior of women held both opportunities and challenges in their career development towards leadership. A woman who has long term goals, is empathetic and a good listner, is wise in the use of resources, and is honest to subordinates is more likely rise to a leadership position. This finding was in line with the arguments of Bass (1999), where personal characteristics which improve team engagement, confidence, and build trust among staff are exhibited among women transformational leaders [32]. Simialry, the finding was in line with Spears (2010) who defined empathy as one of the characteristics of servant leadership [33]. In addition, leadership experiences which were acquired through direct engagement in executing assignments or through lessons from colleagues, family members, and elders it was found assist women to be competent in leadership positions. This finding was in line with the argument of Jyoti and Dev (2015) that infer that leadership skills and competencies were improved through orientation and experience sharing activities [34].

However, women who had negative leadership experiences like those who were micromanaged by their supervisors tend to decline taking on leadership positions. In addition, women who are afraid of experiencing sexual harassment also avoid leadership positions. This challenge for women leaders was documented in three western countries by Folke et al. (2020), where the high risk of sexual harassment reported among lower and mid-level women leaders obstructed their journey to leadership positions [35].

In a developing country like Ethiopia, the burden of household chores is heavy on women. This study’s findings indicated that women who had the support of their spouse, grandparents and peers are more likely assume the roles and responsibilities of senior health leadership positions. Similarly, women that are burdened with household chores and lack social support found it challenging to continue to advance their career development towards leadership. This finding was in line with Bahiru and Mengistu (2018) who confer that women who lack personal and social support from family members were forced to leave their leadership positions due to work overload [36]. In African counties, the leadership career development of female health workers was interrupted due to their engagement with family duties [15, 16].

The lived stories and shared experiences of participants showed that women that received organizational support are well prepared to be leaders in the health system. Some of the organizational support identified by the participants are affirmative actions and recognition. Ely et al. (2011) attest that recognition and affirmation strengthens one’s leadership identity which fuels the search for growth [37]. In line with this finding, in western countries, female health workers disrupted their career development due to lack of a flexible work environment [14].

In a low-income country like Ethiopia, the role of development partners in providing technical, financial, and other resource support is invaluable. Leadership training and mentoring sessions, facilitated with the support of partners build the leadership capacity and competencies of women, and prepare them to be competent for leadership dispensation. This finding aligns with that of Desta et al. (2020) and Argaw et al. (2021), where facilitating leadership training and coaching activities were found to be effective in building the desired competencies [38–40].

In the Ethiopian health system, almost all leadership positions are assumed by men. In this study, the culture and norms of society was indicated as favoring men in terms of their rise to leadership positions. This prevents women from rising to leadership positions. Similarly, because of deep-rooted gender stereotypes held by the community, women have a poor sense of self-image when it comes to managing complex organizations and therefore avoid assuming leadership positions. This finding is supported by Akhtar (2008), Bahiru and Mengistu, (2018), Teede (2019) and Newman et al. (2019) that show that social stereotypes and the institutionalized norms of linking leadership position to men had an influence on the professional career development of women [17–19, 36].

To close the gaps a deliberate gender sensitive health workforce monitoring system is recommended by Kuhlmann et al. (2017) and Rotenstein (2018) [41, 42]. In addition, Dea (2019) strongly recommend emowpering people rather than blaming the system, so that women are able to make decisions, and have the capacity to progress in their sphere of influence in development matters [43, 44]. Silver et al.(2019) reviewed published articles and identified importance of worklife balnance, mentorinsip, preventing secual harassment, social support, and use of technologies to improve work environments [45].

### Strengths and limitations

The historic legacy of inequality and discrimination based on gender creates suffering among women in Ethiopia. Acknowledging the problem leads to the revision of policies, proclamations, and directives [12]. Empowering women and reducing inequalities will benefit families, communities, and the country. This study includes women leaders from three agrarian regions. The indepth interviews were conducted by experienced female public health and social science professionals. In addition, to control the investigators’ bias, all interviewers actively participated in data transcription, coding, and analysis.However, the study does have some limitations as it was conducted in only three agrarian regions of Ethiopia, which means the experiences of women health leaders in at Ministry of Health, general and referral hospitals, private sector, and pastoralist regions were not included. Therefore, the investigator described the context as useful for the interpretation and generalizability of the result of this study only.

## Conclusions

This study delved into participant experiences in the path to leadership positions. The leadership positions were an opportunity in the career development of women who had long-term goals, are known for their empathy and listening skills, and displayed a wise use of resources. In addition, women with positive leadership experiences through delegation and engagement in focusing, inspiring, planning, organizing, directing, and controlling activities were capacitated to assume senior leadership and management positions. Women who had the support of close family members and peers are also more likely to compete for and accept leadership positions. Furthermore, women who had received organizational support in the form of affirmative action, training, development, and recognition also tend to take on leadership positions. However, women who had been influenced in their day-to-day decision making by their supervisors, experienced sexual harassment, and were under the influence of societal norms that favor men are more likely decline leadership positions. Therefore, enhancing the leadership capacity of women, and improving social and organizational support is recommended. In addition, addressing the poor self-image among women, as well as male dominated societal norms at community level is recommended.

## Supplementary Information

**Additional file 1.** Semi-structured interview guide.

## Data Availability

All relevant data are within the paper.

## Abbreviations

LMG: leadership, management, and governance
SNNP: Southern Nations, Nationalities, and Peoples’
USAID: United States Agency for International Development
WHO: World Health Organization
