# Supplementary material for: Exploring the opportunities and challenges of female health leaders in three regional states of Ethiopia: a phenomenological study
Source: BMC Public Health. 2022 Aug 2;22:1471. doi: 10.1186/s12889-022-13871-w (PMC9343224; doi:10.1186/s12889-022-13871-w)
Supplement: Supplementary file 1 — Additional file 1. Semi-structured interview guide. [file 12889_2022_13871_MOESM1_ESM.docx]

**Additional file 1: Semi-structured interview guide**

**Introduction**

This tool has been developed to collect data useful to explore the experiences of women in their path towards assuming health leadership positions and describe the perceived opportunities and challenges of female leaders in health in Ethiopia. USAID Transform: Primary Health Care will collect and analyze the data and synthesize the information for evidence-based decision making by policy makers, program implementers, and future female health leaders. Please, note that the information you give us will be kept confidential and used only to fill information gaps in the health system.

This questionnaire is designed to help conduct in-depth interviews in three regional states of Ethiopia. The tool has three parts: part one is dedicated to capture background information, part two is dedicated as an in-depth interview guide to capture general information, and the third part is dedicated to gather information on career development.

| **Part 1: Background information** | |
| --- | --- |
| **Question** | **Coding categories** |
| 10. Date of assessment | __ __/__ __/__ __ __ __(DD/MM/YYYY) |
| 20. Organization location | Region _____Zone _______Woreda ______PHC________ |
| 30. Age [in full years] | [ ] years |
| 40. Sex | 1. Female |
| 50. Marital status | 1. Single 2. Married 3. Separated/divorced 4. Other (state)-------------------------- |
| 60. Profession |  |
| 70. Overall work experience | [ ] years |
| 80. Work experience in a leadership position | [ ] years |
| 90. Did you participate in leadership, management, and governance trainings | 1. Yes 2. No |
| If yes, (specify) | 1.  2.  3. |

**Part II. In depth interview guide**

Participants of this study will be asked the following questions:

1. What have you experienced as a woman on your path toward health leadership?
   1. In what ways do you think gender has affected your career path?
   2. Does it continue to affect how you lead in your current position?
2. What contexts or situations have typically influenced or affected your experiences as a woman in a leadership position?
   1. What have been some of your motivating factors from the start of your career until now? E.g. training, opportunity, mentoring/coaching, quotas, general discussion.
   2. What enabling factors do you think were the most significant in getting you to where you are? What did you have to overcome, if anything?
   3. Do you credit any individual, institution, or program with supporting you on your path to leadership?
   4. What are some of the biggest crisis situations or critical events you’ve had to handle?
   5. What (if any) benefits or advantages do women bring to leadership and organizations, in your opinion?
   6. What do you think are the best tools to increase the number of women in leadership positions? Recommendations.
